# High-Level Systematic Ab Initio Comparison of Carbon- and Silicon-Centered S_N_2 Reactions

**DOI:** 10.1021/acs.jpca.1c07574

**Published:** 2021-10-28

**Authors:** Attila Á. Dékány, Gyula Z. Kovács, Gábor Czakó

**Affiliations:** MTA-SZTE Lendület Computational Reaction Dynamics Research Group, Interdisciplinary Excellence Centre and Department of Physical Chemistry and Materials Science, Institute of Chemistry, University of Szeged, Rerrich Béla tér 1, Szeged H-6720, Hungary

## Abstract

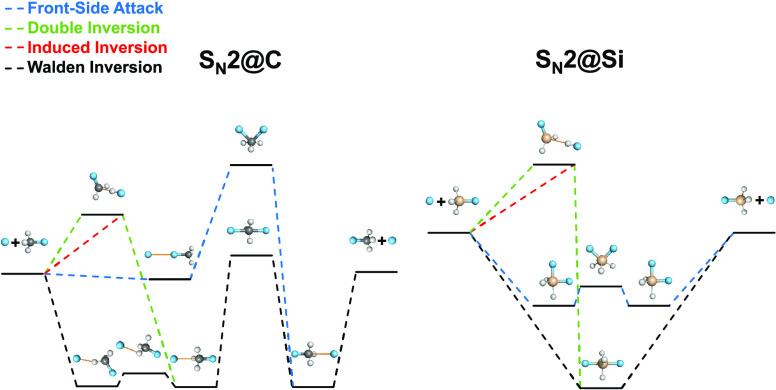

We characterize the stationary points along the Walden inversion, front-side attack, and double-inversion pathways of the X^–^ + CH_3_Y and X^–^ + SiH_3_Y [X, Y = F, Cl, Br, I] S_N_2 reactions using chemically accurate CCSD(T)-F12b/aug-cc-pV*n*Z [*n* = D, T, Q] levels of theory. At the carbon center, Walden inversion dominates and proceeds via prereaction (X^–^···H_3_CY) and postreaction (XCH_3_···Y^–^) ion-dipole wells separated by a usually submerged transition state (X–H_3_C–Y)^−^, front-side attack occurs over high barriers, double inversion is the lowest-energy retention pathway for X = F, and hydrogen- (F^–^···HCH_2_Y) and halogen-bonded (X^–^···YCH_3_) complexes exist in the entrance channel. At the silicon center, Walden inversion proceeds through a single minimum (X–SiH_3_–Y)^−^, the front-side attack is competitive via a usually submerged transition state separating pre- and postreaction minima having X–Si–Y angles close to 90°, double inversion occurs over positive, often high barriers, and hydrogen- and halogen-bonded complexes are not found. In addition to the S_N_2 channels (Y^–^ + CH_3_X/SiH_3_X), we report reaction enthalpies for proton abstraction (HX + CH_2_Y^–^/SiH_2_Y^–^), hydride substitution (H^–^ + CH_2_XY/SiH_2_XY), XH···Y^–^ complex formation (XH···Y^–^ + ^1^CH_2_/^1^SiH_2_), and halogen abstraction (XY + CH_3_^–^/SiH_3_^–^ and XY^–^ + CH_3_/SiH_3_).

## 1 Introduction

Since Walden’s discovery in 1896,^1^ bimolecular nucleophilic substitution (S_N_2) reactions at a tetrahedral carbon center have been widely studied both experimentally and theoretically.^2−35^ The prototypes of these reactions are X^–^ + CH_3_Y → Y^–^ + CH_3_X, where X, Y = F, Cl, Br, I. The X^–^ + CH_3_Y S_N_2 reactions usually proceed with Walden inversion on a double-well potential along the collinear X–C–Y arrangement via a prereaction ion-dipole complex (X^–^···H_3_CY), a central transition state (X–CH_3_–Y)^−^, and a postreaction ion-dipole complex (XCH_3_···Y^–^). In addition to the well-known Walden-inversion mechanism, it was earlier recognized^2^ that S_N_2 reactions may occur via a high-energy front-side attack transition state (XYCH_3_)^−^, where the XCY angle is around 80°.^11,20,22,23,25,26,36−38^ Furthermore, recent studies revealed that hydrogen- (X^–^···HCH_2_Y)^15,18,20^ and halogen-bonded (X^–^···YCH_3_)^24,29,33^ complex formations may also play key roles in carbon-centered S_N_2 reactions. Moreover, reaction dynamics simulations uncovered a double-inversion pathway for the F^–^ + CH_3_Cl S_N_2 reaction, which provides products with retention of the initial configuration via double-inversion (FH···CH_2_Cl^–^) and Walden-inversion (F–CH_3_–Cl)^−^ transition states.^21^ Since the discovery of double inversion in 2015,^21^ this mechanism has been identified in several other S_N_2 reactions both in the gas^22,23,26,27^ and solution^39,40^ phases. In addition to the widely studied carbon-centered systems, the S_N_2 reaction can also occur at the silicon center.^41−56^ Even though silicon is below carbon in the periodic table, it is well established in the literature^41−56^ that silicon-centered S_N_2 reactions are quite different from their carbon-centered analogues. The X^–^ + SiH_3_Y reactions can be characterized by a single-well Walden-inversion potential featuring a deep minimum corresponding to the penta-covalent (X–SiH_3_–Y)^−^ complex. Furthermore, due to the large size of the Si atom, at the silicon center, the front-side attack transition states (XYSiH_3_)^−^ are also usually submerged, opening barrierless retention pathways that may compete with Walden inversion.^41,47−50^ Despite the interesting features of silicon-centered S_N_2 reactions, these systems are less studied than the carbon-centered analogues. In the case of the X^–^ + SiH_3_Y-type reactions, mostly the Cl^–^ + SiH_3_Cl (refs (41, 43−50, 53, 54, 56)) identity process is investigated in addition to the few studies on F^–^ + SiH_3_F (refs (41, 48, 56)). The theoretical studies on these reactions usually used density functional theory with triple-zeta basis sets to characterize the stationary points,^43−47,50,54,56^ and in some cases,^43,49,50^ the energies are refined by the CCSD(T)/aug-cc-pVQZ level of theory.

In the present study, we report a comprehensive ab initio investigation of the identity and nonidentity X^–^ + SiH_3_Y S_N_2 reactions with X, Y = F, Cl, Br, I and compare them with the carbon-centered analogues. For the first time in the case of the Si-centered systems, we employ the explicitly correlated CCSD(T)-F12b method^57^ to obtain benchmark structures, vibrational frequencies, and relative energies of the stationary points. In addition to the back-side and front-side attack, we also explore the possibility of double inversion in Si-centered S_N_2 reactions. Furthermore, we determine the reaction enthalpies of several alternative product channels obtained by, for example, proton and halogen abstractions. These results may shape our fundamental knowledge of model ion–molecule reactions at carbon and silicon centers and guide future global potential energy surface developments and experimental and theoretical dynamics studies. The computational details are summarized in Section 2, the results are described and discussed in Section 3, and the paper ends with summary and conclusions in Section 4.

## 2 Computational Details

We determined optimized geometries and harmonic vibrational wavenumbers of the minima and saddle points of carbon- and silicon-centered X^–^ + CH_3_Y/SiH_3_Y [X, Y = F, Cl, Br, I] S_N_2 reactions using the explicitly correlated coupled-cluster singles, doubles, and perturbative triples CCSD(T)-F12b method^57^ with the augmented correlation-consistent aug-cc-pV*n*Z (*n* = D, T) basis sets.^58^ In the case of Br and I, the corresponding aug-cc-pV*n*Z-PP basis sets are used employing effective core potentials considering relativistic effects.^59^ Our highest-level electronic energy computations are performed using the aug-cc-pVQZ(-PP) basis sets^58,59^ on the aug-cc-pVTZ(-PP) geometries. For open-shell systems (XY^–^ and CH_3_/SiH_3_), the unrestricted UCCSD(T)-F12b method^60^ is used based on restricted open-shell Hartree–Fock^61^ orbitals. All computations are carried out using the MOLPRO program package.^62^

## 3 Results and Discussion

**Figure 1:**
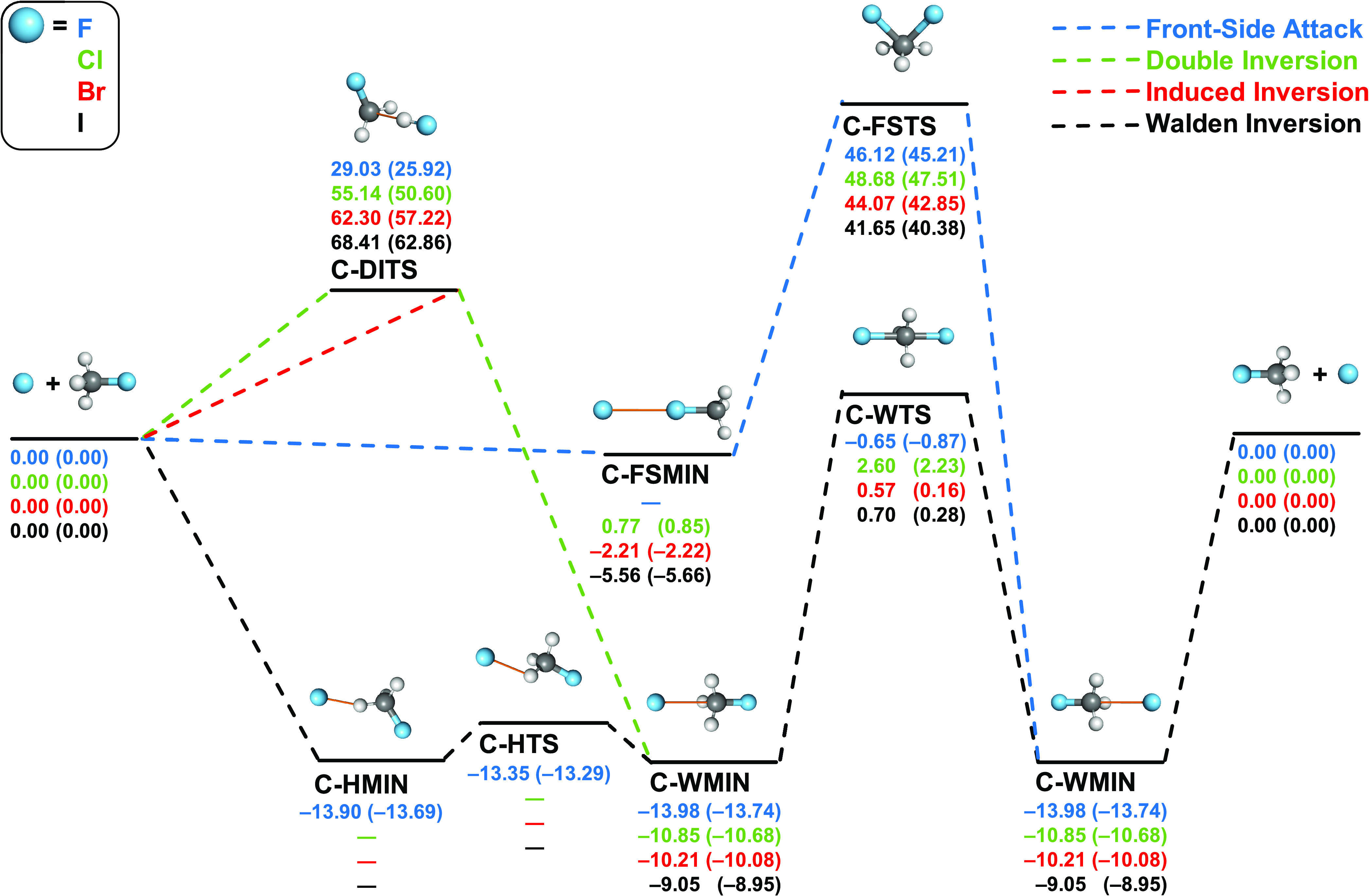
Pathways of X^–^ + CH_3_X (X = F, Cl, Br, I) S_N_2 reactions showing classical (adiabatic) relative energies in kcal/mol obtained at the CCSD(T)-F12b/aug-cc-pVQZ(-PP)//CCSD(T)-F12b/aug-cc-pVTZ(-PP) level of theory.

**Figure 2:**
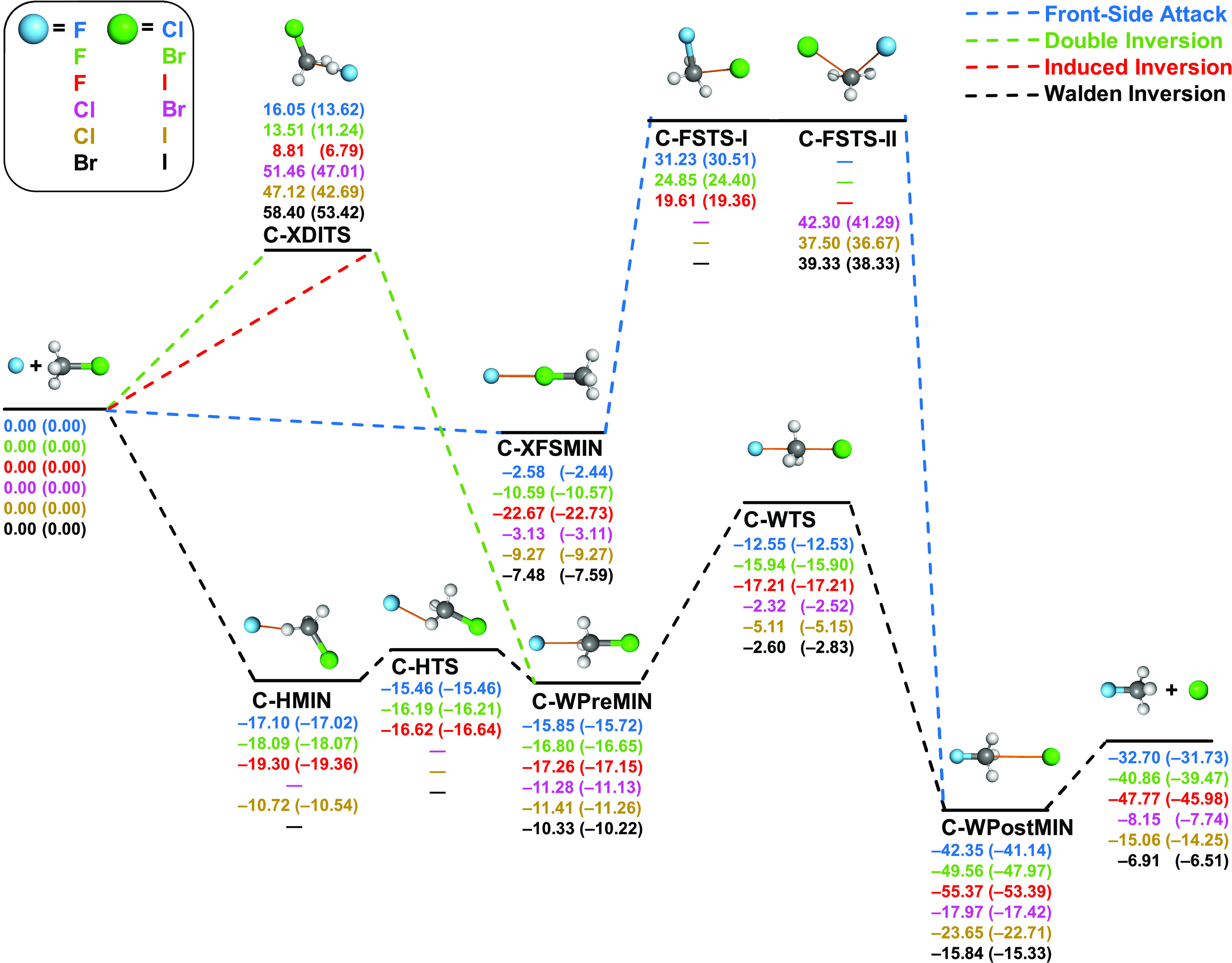
Pathways of X^–^ + CH_3_Y (X = F, Cl, Br; Y = Cl, Br, I) S_N_2 reactions showing classical (adiabatic) relative energies in kcal/mol obtained at the CCSD(T)-F12b/aug-cc-pVQZ(-PP)//CCSD(T)-F12b/aug-cc-pVTZ(-PP) level of theory.

Figures 1 and 2 schematically present the most important pathways of the gas-phase identity and nonidentity halide ion and methyl-halide S_N_2 reactions, respectively, in which the atomic number of the nucleophile is either equal to or smaller than the atomic number of the substituent. The considered pathways include Walden inversions, front-side attacks, induced inversions, and double inversions.

### 3.1 Pathways of Carbon-Centered S_N_2 Reactions

According to Figure 1, the energetically most-accessible Walden-inversion pathways of the identity reactions have low barriers in the case of Cl, Br, and I, whereas a small negative energy barrier is found for F (see C-WTS), surrounded by a symmetric double well in all cases. The ion-dipole complexes (C-WMIN) have decreasing stability from F to I with dissociation energies in the 9–14 kcal/mol range. Furthermore, a hydrogen-bonded low-energy minimum (C-HMIN) and a saddle point (C-HTS) connecting C-HMIN and C-WMIN are present only for F. The energies of C-HMIN and C-WMIN are the same within 0.1 kcal/mol for the F^–^ + CH_3_F reaction. In contrast to the Walden-inversion pathway, the front-side attack mechanism goes through high-energy saddle points (C-FSTS) with barrier heights varying in the 40–46 kcal/mol range for all of the four cases. Except for F (and Cl), a front-side collision can yield a halogen-bonded complex, C-FSMIN, with *D*_0_ dissociation energies of 2.22 and 5.66 kcal/mol for Br and I, respectively. For X = F, this stationary point cannot be found and for Cl and C-FSMIN has positive energy relative to the reactants. By comparing the relative energies of C-FSTSs and double-inversion transition states (C-DITSs), we can conclude that the double inversion is more accessible than the front-side pathway for the X = F identity reaction but less favorable for the other three cases.

Figure 2 shows the corresponding pathways for the exothermic nonidentity carbon-centered S_N_2 reactions. Similar to the identity reactions, the back-side attack leads to a transition state, C-WTS, surrounded by a double well, but there are notable differences. Energetically, the F/Cl, F/Br, and F/I Walden-inversion transition states have deeper negative relative energies between −12.55 and −17.21 kcal/mol classically, whereas, for Cl/Br, Cl/I, and Br/I, the corresponding values are still negative, varying between −2.32 and −5.11 kcal/mol. The relative energies of C-WPostMINs also form two groups, deeper minima—relative to the reactants—for F/Cl, F/Br, and F/I and shallower negative energy minima for the other cases in accord with the trends in the exothermicities. The hydrogen-bonded low-energy saddle points, C-HTSs, consistent with the identity reactions are obtained only for the X = F reactions. In the cases where C-HTSs exist, they are below the corresponding C-WTSs and the hydrogen-bonded minima (C-HMINs) are deeper than the corresponding C-WPreMINs. Interestingly, the hydrogen-bonded Cl/I minimum can also be obtained, possibly, due to the low electronegativity of the I substituent; however, this C-HMIN is slightly above the corresponding C-WPreMIN. C-XFSMINs, similar to the C-FSMIN (in the identity reactions) structures, are minima with negative relative energies. In most cases, C-XFSMINs are significantly shallower than the corresponding C-HMINs and C-WPreMINs, except for F/I, where C-XFSMIN with *D*_e_(*D*_0_) of 22.67(22.73) kcal/mol is the deepest minimum in the entrance channel. Thus, for F/I, the deep C-XFSMIN steers the reactants away from the reactive back-side attack configurations, thereby making the F^–^ + CH_3_I S_N_2 reaction indirect as shown in the previous simulations and experiments.^24,29^ The nonidentity front-side attack transition states of F/Cl, F/Br, and F/I (C-FSTS-Is) compared to Cl/Br, Cl/I, and Br/I C-FSTS-IIs are very different geometrically. The former TSs possess the *C_s_* point-group symmetry with four atoms (C, H, and two halogens) in its mirror planes and the latter three structures resembling more the TSs of the identity reaction counterparts with *C*_1_ symmetry. Energetically, the double-inversion TSs (C-XDITSs) are more accessible to F-containing reactions than C-FSTS-Is, whereas the relative energies of Cl/Br, Cl/I, and Br/I C-XDITSs are higher than the relative energies of the corresponding C-FSTS-II saddle points. The lowest-energy retention pathways are found for the F/I reaction with classical (adiabatic) double-inversion and front-side attack barrier heights of 8.81(6.79) and 19.61(19.36) kcal/mol, respectively.

### 3.2 Pathways of Silicon-Centered S_N_2 Reactions

**Figure 3:**
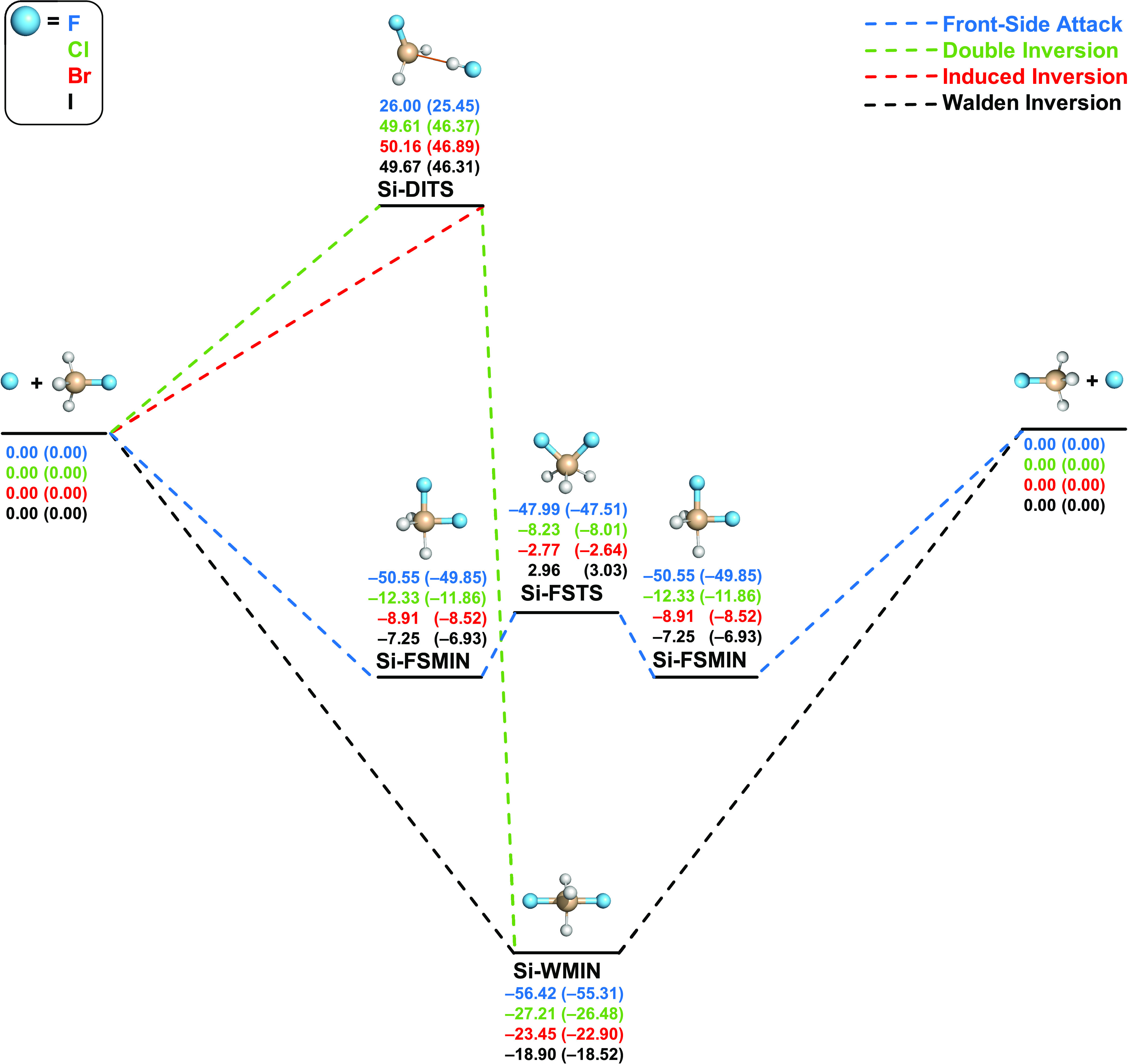
Pathways of X^–^ + SiH_3_X (X = F, Cl, Br, I) S_N_2 reactions showing classical (adiabatic) relative energies in kcal/mol obtained at the CCSD(T)-F12b/aug-cc-pVQZ(-PP)//CCSD(T)-F12b/aug-cc-pVTZ(-PP) level of theory.

**Figure 4:**
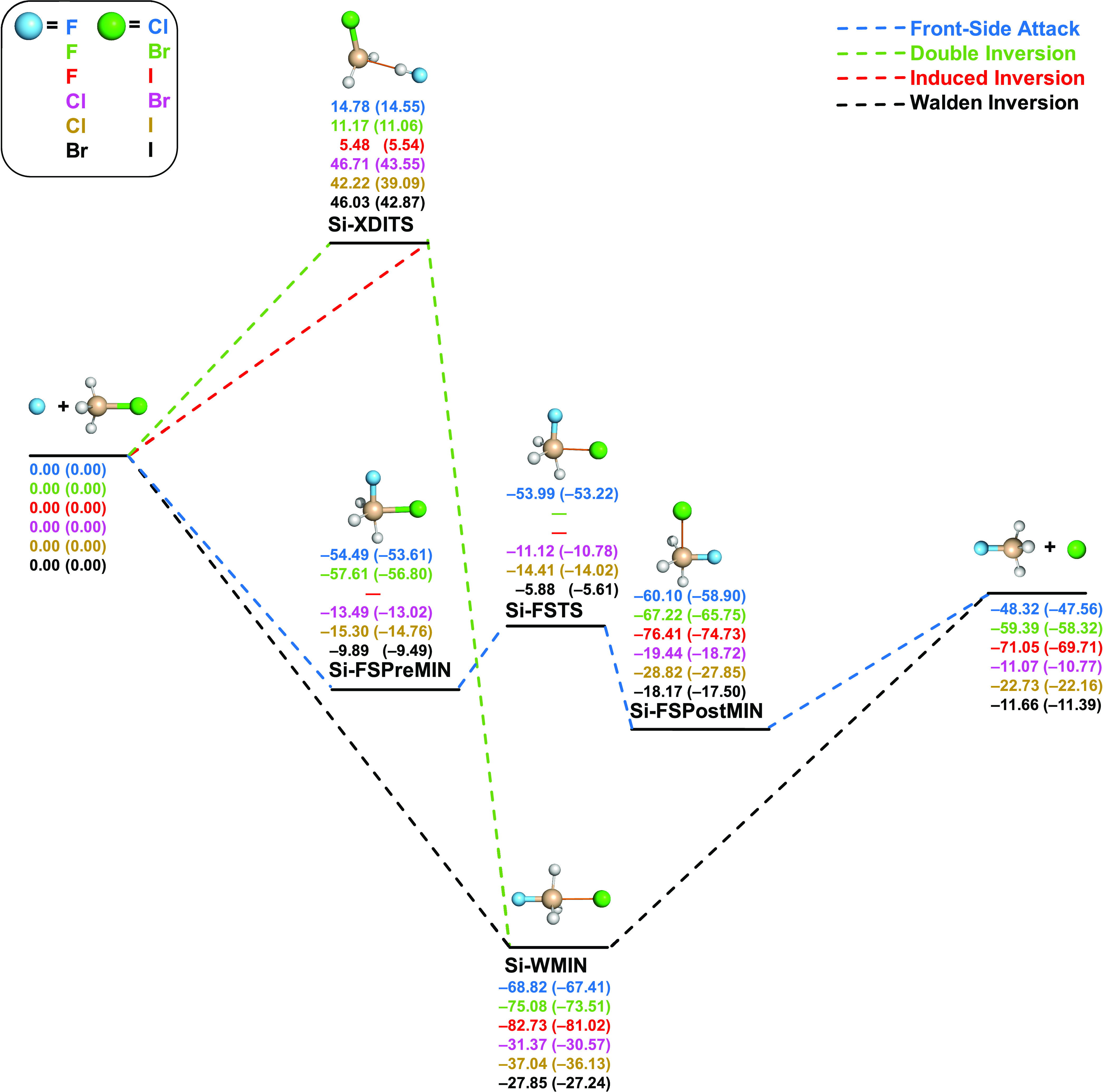
Pathways of X^–^ + SiH_3_Y (X = F, Cl, Br; Y = Cl, Br, I) S_N_2 reactions showing classical (adiabatic) relative energies in kcal/mol obtained at the CCSD(T)-F12b/aug-cc-pVQZ(-PP)//CCSD(T)-F12b/aug-cc-pVTZ(-PP) level of theory.

As shown in Figures 3 and 4, the possible pathways of the gas-phase halide ion and silyl-halide S_N_2 identity and nonidentity reactions are comparable to the carbon-centered counterparts, but the corresponding back- and front-side attack pathways and the underlying potential energy surfaces are qualitatively different.

In contrast to the carbon-centered reactions, the Walden-inversion pathways of the X^–^ + SiH_3_Y S_N_2 reactions do not go through transition states, and only a deep minimum connects the reactants and products with depths between 18.90 and 56.42 kcal/mol for identity reactions and from 27.85 to 82.73 kcal/mol classically for nonidentity reactions. The minima are significantly deeper for X = F (*D*_e_ = 56.42, 68.82, 75.08, 82.73 kcal/mol for Y = F, Cl, Br, I, respectively) than in the case of the other nucleophiles (*D*_e_ = 18.90–37.04 kcal/mol). On the other hand, the front-side attacks on the Si center feature double-well potentials with saddle points (Si-FSTSs) separating minima (Si-FSMINs) symmetrically in the identity reactions and Si-FSPreMINs and Si-FSPostMINs for the nonidentity reactions. The TSs of the front-side attacks are geometrically like carbon-centered C-FSTSs in the identity reactions and comparable to C-FSTS-IIs of the nonidentity reactions. Energetically, the silicon-centered front-side attack pathways are accessible at lower energies than C-FSTSs, as Si-FSTSs are submerged in almost all cases, except for the I identity reaction, where the barrier height is a low positive value (2.96 kcal/mol classically). In some cases, especially for X = F, the Walden-inversion pathway is just slightly below the corresponding front-side attack retention path; thus, one may expect interesting competition between inversion and retention for silicon-centered S_N_2 reactions, which may be investigated by dynamics simulations in the near future. The most similar pathways of the C- and Si-centered reactions are double inversions. The silicon-centered Si-DITSs (identity reactions) and Si-XDITSs (nonidentity reactions) are geometrically and energetically similar to the corresponding carbon-centered TSs. However, unlike for the F^–^ + CH_3_Y systems, at the Si center, the positive double-inversion barriers are always above the corresponding Si-FSTSs.

### 3.3 Carbon-Centered Stationary Points

**Figure 5:**
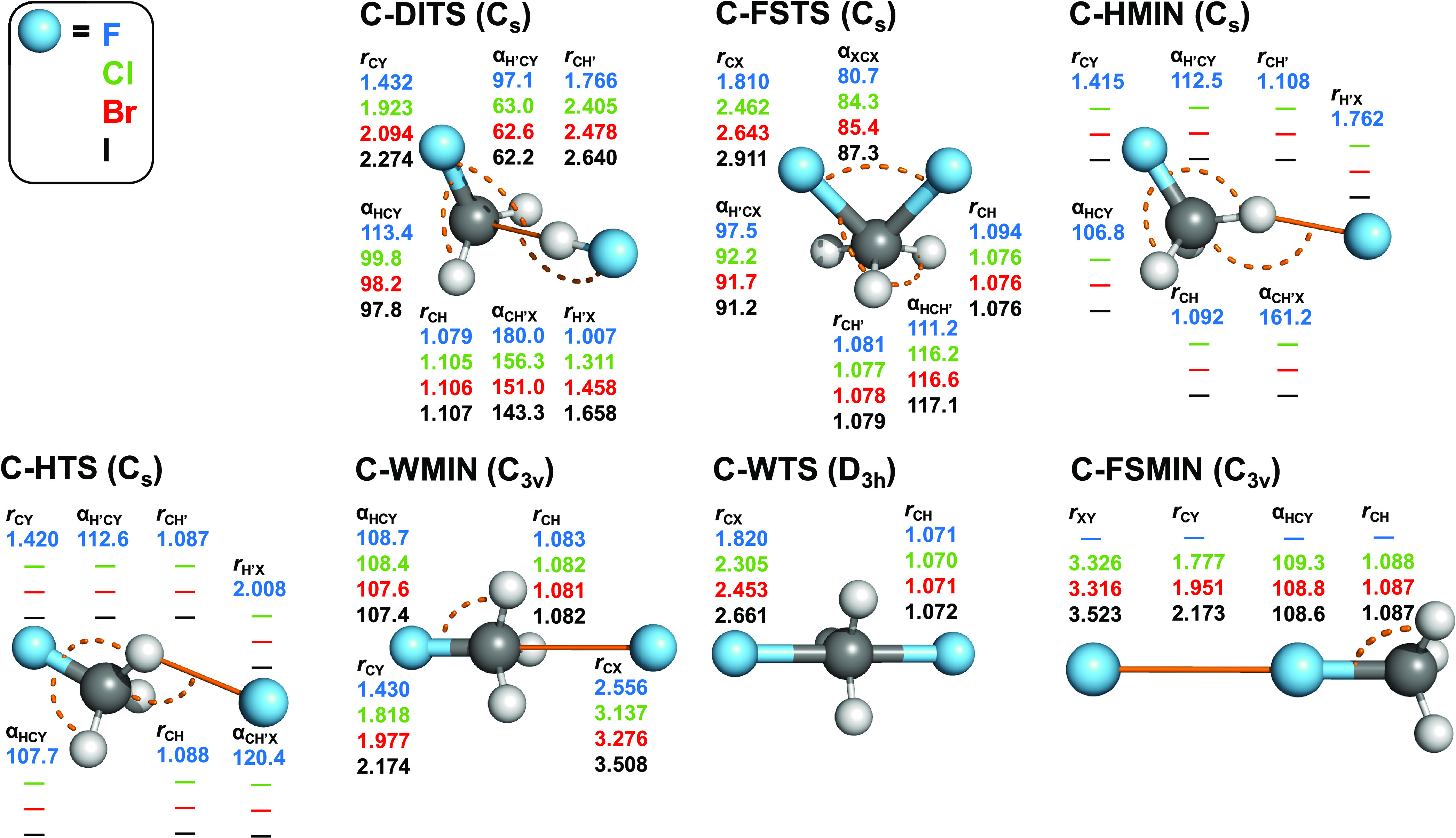
Structures and point groups of minima and saddle points of the X^–^ + CH_3_X (X = F, Cl, Br, I) reactions optimized at the CCSD(T)-F12b/aug-cc-pVTZ(-PP) level of theory. Bond lengths and angles are given in angstroms and degrees, respectively. In the case of *C_s_* structures, hydrogen atoms lying in the mirror plane are denoted by H′. In the subscripts, X and Y denote the nucleophile and substituent, respectively. X and Y are the same halogens but in different chemical environments.

**Figure 6:**
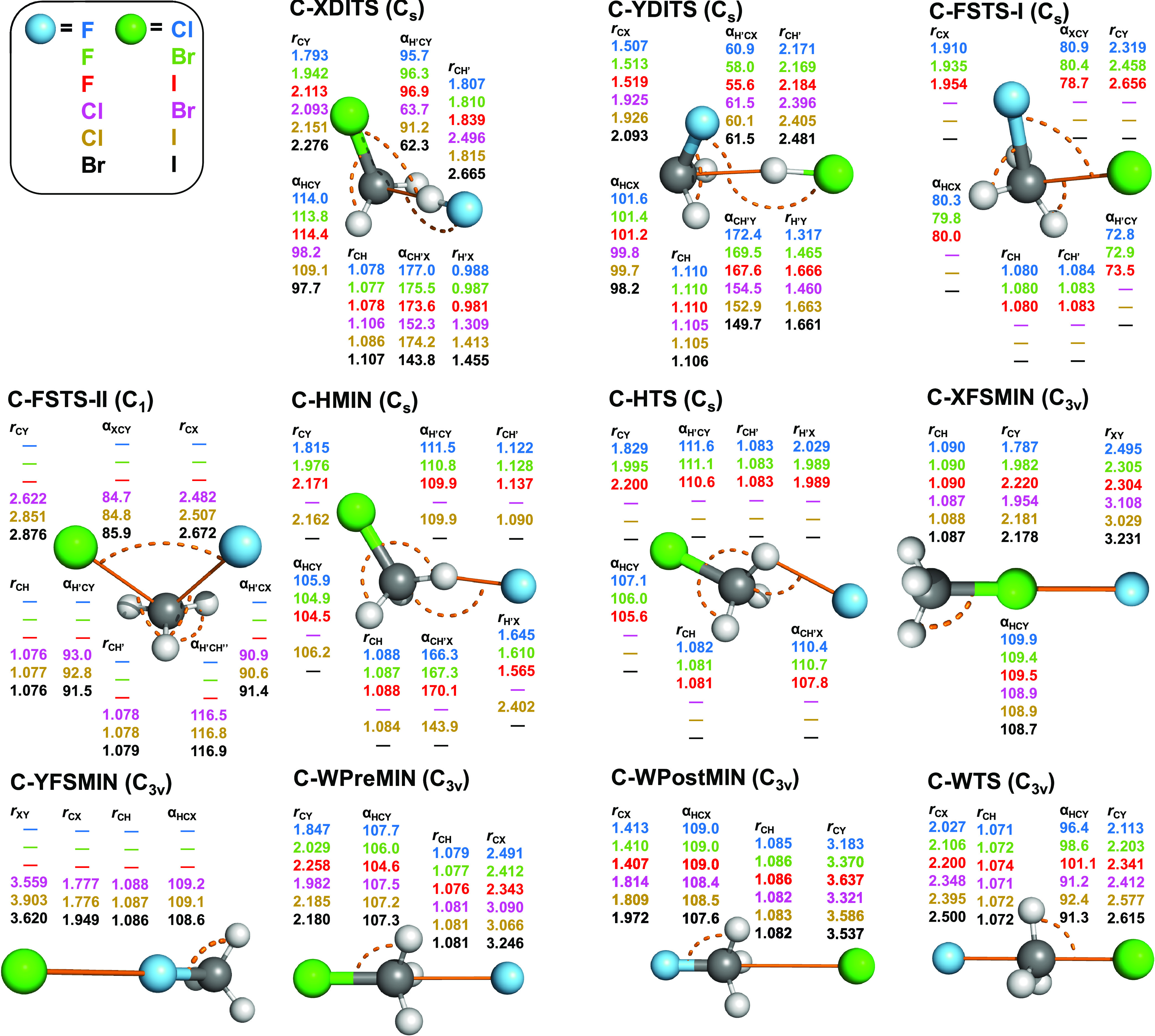
Structures and point groups of minima and saddle points of the X^–^ + CH_3_Y (X = F, Cl, Br; Y = Cl, Br, I) reactions optimized at the CCSD(T)-F12b/aug-cc-pVTZ(-PP) level of theory. Bond lengths and angles are given in angstroms and degrees, respectively. In the case of *C_s_* structures, hydrogen atoms lying in the mirror plane are denoted by H′.

Figures 5 and 6 show the most important benchmark structural parameters of the stationary points (minima and transition states) of the potential energy surfaces of the carbon-centered identity- and nonidentity reactions, respectively. Contrary to Figures 1 and 2, C-YDITSs and C-YFSMINs, the endergonic nonidentity reaction counterparts of C-XDITSs and C-XFSMINs, respectively, are also presented in Figure 6.

As shown in Figure 5, all identity-reaction double-inversion TSs (C-DITSs), front-side attack TSs (C-FSTSs), and hydrogen-bonded C-HMIN and TS (C-HTSs) of the X = F reaction have *C_s_* point-group symmetry. Front-side attack halogen-bonded complexes, C-FSMINs, possess a 3-fold *C*_3*v*_ symmetry. The point group of the Walden minima, C-WMINs, is also *C*_3*v*_ and the most symmetric structures are the C-WTSs belonging to *D*_3*h*_.

Figure 6 shows the stationary points of the nonidentity reactions. The point-group symmetries of the double-inversion TSs (C-XDITSs and C-YDITSs) are, like in the identity reactions, all *C_s_*. However, the F/Cl, F/Br, and F/I front-side TSs (C-FSTS-Is) are with the same point-group symmetry, *C_s_*, as the identity reaction counterparts, and these geometries are structurally very different. In the case of identity reactions, the plane of reflection goes through the C atom and one of the hydrogens, whereas, in the case of the nonidentity C-FSTS-Is, the two halogens are in the plane as well. The true counterparts of the identity reaction C-FSTSs are C-FSTS-IIs, found for Cl/Br, Cl/I, and Br/I, with *C*_1_ point-group symmetry. The symmetries of C-HMINs and C-HTSs are comparable to the corresponding minimum and the transition state found for the F identity reaction. Walden transition states (C-WTSs) have *C*_3*v*_ symmetry with collinear X–C–Y arrangements and structurally (and energetically) more like preminimum structures (C-WPreMINs) than C-WPostMINs.

Considering the structural parameters shown in Figures 5 and 6, we can observe that at C-WMIN, C-WPreMIN, and C-WPostMIN, the methyl-halide unit is only slightly perturbed and the C···X and C···Y distances are around 2.3–2.6, 3.1–3.2, 3.3–3.4, and 3.5 Å for X/Y = F, Cl, Br, and I, respectively. C-HMIN and C-HTS present for X = F, with H′···F distances of around 1.6–1.8 Å (HMIN) and 2.0 Å (HTS) and CH′F angles of 160–170° (HMIN), and 108–120° (HTS), where H′ denotes the hydrogen-bonded H atom. For the identity reactions, the C–X distances are stretched by 0.4–0.5 Å at C-WTS relative to the corresponding bond length in the CH_3_X molecules. For the nonidentity reactions, the trends are similar but subtle differences can be observed; for example, the C–F distances are stretched by 0.6–0.8 Å. At the front-side minima, the CY···X atoms are collinear and the Y···X distance is around 2.3 Å for X/Y = F/Br and F/I and 2.5 Å for X/Y = F/Cl, whereas the halogen bond lengths are between 3 and 4 Å for X = Cl, Br, and I, in accord with the highest stability of the F/Br and F/I complexes. At the front-side attack TSs, the C–X and C–Y bonds are usually 0.1–0.2 Å longer than the corresponding distances in C-WTSs, except for the C–F bonds, which are shorter by 0.1–0.2 Å. Furthermore, the X–C–Y angles are in the 78–88° range for the front-side TSs, whereas the X–C–Y atoms are exactly collinear in C-WTSs. At the DITSs, the bond length of the HX fragment is close to the corresponding value in the HX molecule and the C···H interfragment distance is around 1.8 Å for X = F and 2.4–2.7 Å for X = Cl, Br, I, except for Cl/I.

### 3.4 Silicon-Centered Stationary Points

**Figure 7:**
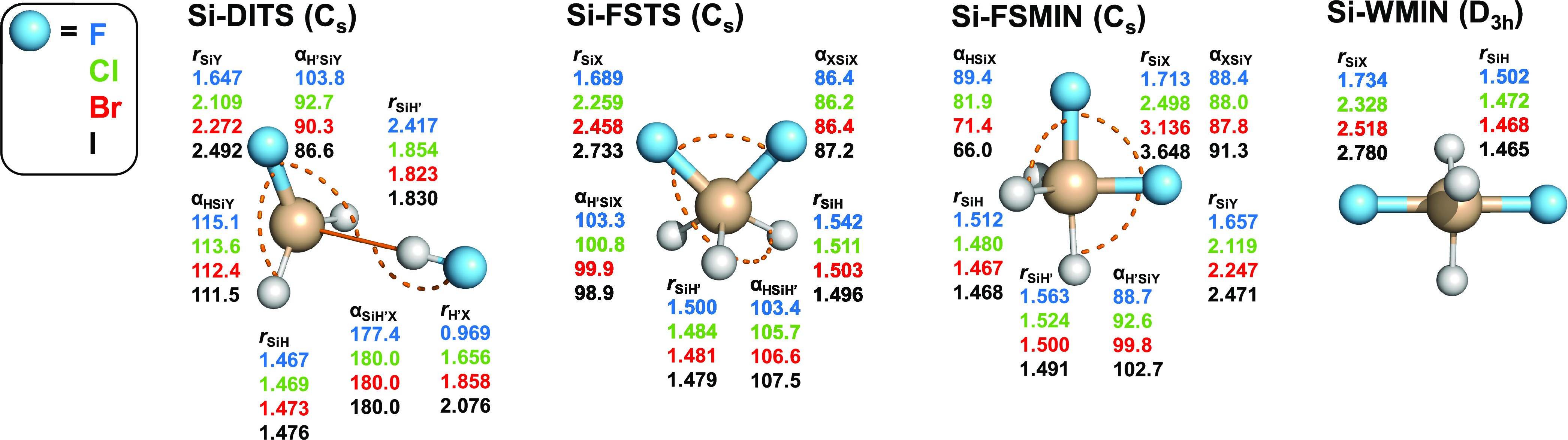
Structures and point groups of minima and saddle points of the X^–^ + SiH_3_X (X = F, Cl, Br, I) reactions optimized at the CCSD(T)-F12b/aug-cc-pVTZ(-PP) level of theory. Bond lengths and angles are given in angstroms and degrees, respectively. In the case of *C_s_* structures, hydrogen atoms lying in the mirror plane are denoted by H′. In the subscripts, X and Y denote the nucleophile and substituent, respectively. X and Y are the same halogens but in different chemical environments.

**Figure 8:**
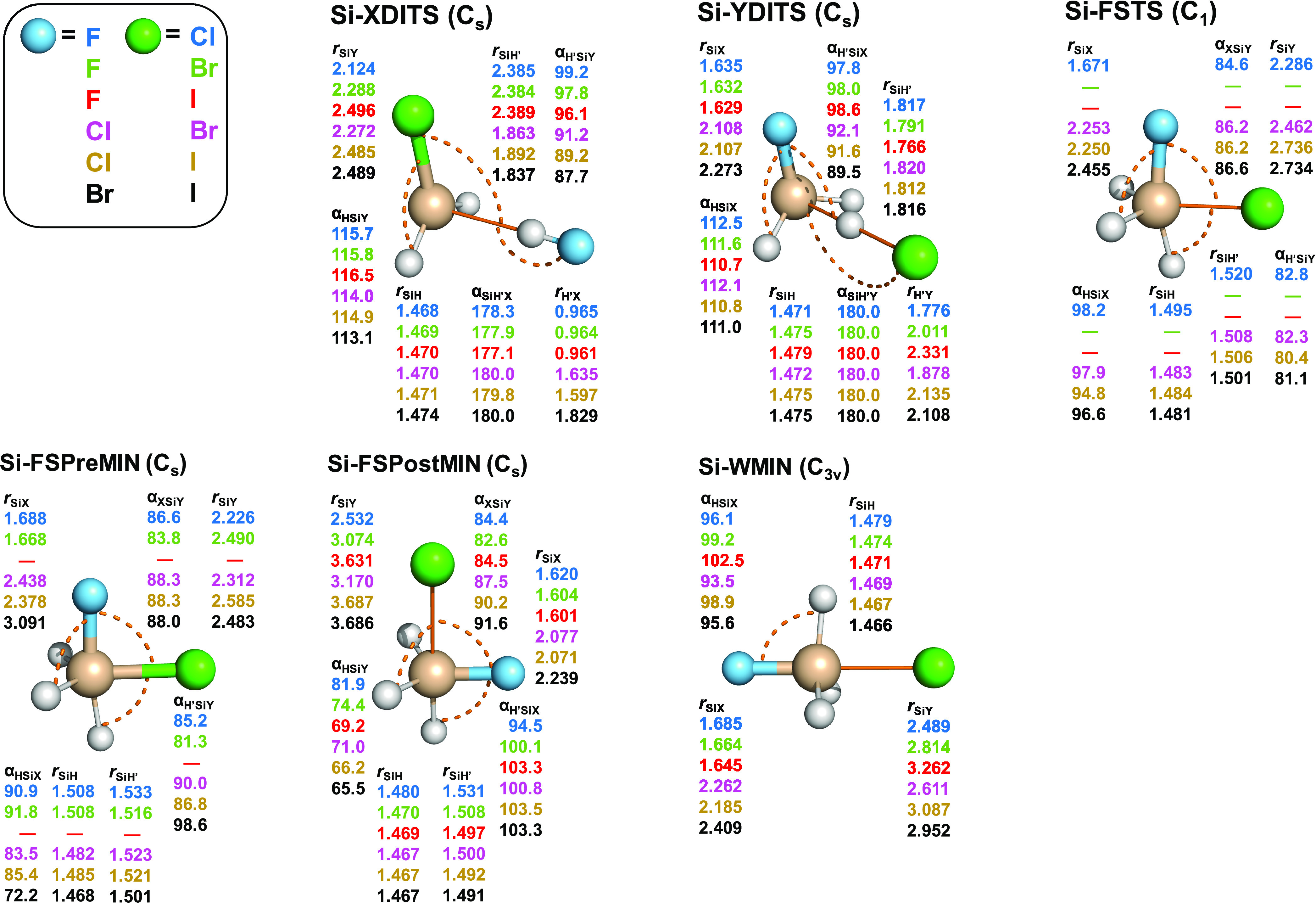
Structures and point groups of minima and saddle points of the X^–^ + SiH_3_Y (X = F, Cl, Br; Y = Cl, Br, I) reactions optimized at the CCSD(T)-F12b/aug-cc-pVTZ(-PP) level of theory. Bond lengths and angles are given in angstroms and degrees, respectively. In the case of *C_s_* structures, hydrogen atoms lying in the mirror plane are denoted by H′.

The benchmark stationary-point structures for the identity and nonidentity silicon-centered S_N_2 reactions are shown in Figures 7 and 8, respectively. In the case of the identity reactions, the structure of Si-WMINs is very similar to that of C-WTSs, i.e., both have a *D*_3*h*_ symmetry and the Si–X and C–X distances agree within about 0.1 Å. For the nonidentity reactions, the symmetries of Si-WMINs and C-WTSs are also the same (*C*_3*v*_); however, unlike the reactant-like C-WTSs, the Si-WMINs are clearly product-like as the H–Si–X angle is greater than 90° and the Si–Y/Si–X distance ratios are significantly larger than the corresponding C–Y/C–X values. Hydrogen- and halogen-bonded complexes are not found for the X^–^ + SiH_3_Y systems but there are front-side minima (Si-FSMIN, Si-FSPreMIN, and Si-FSPostMIN), which are not present for the C-centered reactions. Si-FSMINs of the identity reactions have *C_s_* symmetry, where XXSiH′ atoms are in the symmetry plane, the X–Si–X angle is close to 90°, and the Si–H′ bond length is slightly stretched relative to the other two SiH distances. Si-FSPreMINs and Si-FSPostMINs of the nonidentity reactions have similar *C_s_* structures as Si-FSMINs, but the Si–X distances are longer in Si-FSPreMINs than in Si-FSPostMINs, and the reverse is true for the Si–Y distances. FSTSs of the identity reactions have similar *C_s_* structures at C and Si centers but they differ from Si-FSMINs because at FSTSs only the central atom and one of the hydrogen atoms are in the *C_s_* plane. In the case of the nonidentity reactions, Si-FSTSs are nonsymmetric like the Cl/Br, Cl/I, and Br/I carbon-centered analogues. The X–Si–Y angles are in the 84–88° range for all of the Si-FSTS structures, similar to the C-centered FSTSs. The C- and Si-centered DITSs have similar *C_s_* geometries; however, the C/Si–H′–X distances are significantly different in some cases. On the one hand, for X = F, the H′–F distance is close to the bond length of the HF molecule in both the C- and Si-centered cases, but the Si–H′ distance (∼2.4 Å) is substantially longer than the C–H′ distance (∼1.8 Å). On the other hand, for X = Cl, Br, I, the H′–X distances are stretched by about 0.4 Å relative to the corresponding distances of C-DITSs and HX molecules. Furthermore, the Si–X′ distances are only around 1.8–1.9 Å, whereas the corresponding C–X′ values are usually in the 2.4–2.7 Å range.

### 3.5 Reaction Enthalpies for Various Product Channels

We determined the reaction enthalpies corresponding to the various product channels of the X^–^ + CH_3_Y and X^–^ + SiH_3_Y reactions as shown in Tables 1 and 2, respectively. In addition to the S_N_2 channel leading to Y^–^ + CH_3_X/SiH_3_X, we consider the proton abstraction (HX + CH_2_Y^–^/SiH_2_Y^–^), hydride substitution (H^–^ + CH_2_XY/SiH_2_XY), XH···Y^–^ complex-formation (XH···Y^–^ + ^1^CH_2_/^1^SiH_2_), and halogen-abstraction (XY + CH_3_^–^/SiH_3_^–^ and XY^–^ + CH_3_/SiH_3_) channels. For the X^–^ + CH_3_Y reactions, the S_N_2 channels are thermoneutral (X = Y) or exothermic and all other product channels are endothermic if the atomic number of X is less than or equal to that of Y. For X, Y = Cl, Br, I, halogen abstraction forming XY^–^ + CH_3_ has the lowest endothermicity. For X = F, proton abstraction is thermodynamically more favored than FY^–^ formation, whereas for X = Cl, Br, I, proton abstraction is the second lowest-energy endothermic channel. Then, XH···Y^–^ formation opens usually with about 15 kcal/mol higher endothermicity than proton abstraction. Hydride substitutions are usually highly endothermic and have larger reaction enthalpies than the corresponding XH···Y^–^ + ^1^CH_2_ channels except for X/Y = F/F. In all cases, except for Br/I and I/I, the XY + CH_3_^–^ channels are the most endothermic; however, these product asymptotes correspond to excited singlet electronic states above the two doublet products (XY^–^ + CH_3_), which can also be formed on a singlet potential energy surface. Furthermore, we note that even if the ground electronic state of CH_2_ is triplet, we consider here the singlet XH···Y^–^ + ^1^CH_2_ products because these can be obtained with adiabatic dynamics.

**Table 1:** Classical and Zero-Point Vibrational Energy Corrected (Adiabatic) Energies of X^–^ + CH_3_Y → P + Q (X, Y = F, Cl, Br, I) Reaction Channels Obtained at the CCSD(T)-F12b/aug-cc-pVQZ(-PP)//CCSD(T)-F12b/aug-cc-pVTZ(-PP) Level of Theory in kcal/mol^a^

Q	Y^–^	HX	H^–^	XHY^–^	XY	XY^–^
P	CH_3_X	CH_2_Y^–^	CH_2_XY	^1^CH_2_	CH_3_^–^	CH_3_
F/F	0.0(0.0)	42.2(37.8)	46.3(42.3)	55.3(47.5)	155.0(149.4)	87.0(81.5)
Cl/Cl	0.0(0.0)	68.6(63.0)	91.4(86.1)	83.5(73.6)	112.6(107.5)	57.8(53.1)
Br/Br	0.0(0.0)	74.6(68.7)	100.7(95.1)	88.7(78.9)	104.6(99.6)	46.4(41.9)
I/I	0.0(0.0)	78.8(72.6)	108.3(102.4)	95.8(85.9)	94.7(90.0)	37.0(32.8)
F/Cl	–32.7(−31.7)	28.7(24.8)	54.5(50.3)	43.6(37.6)	103.8(99.0)	52.7(48.1)
F/Br	–40.9(−39.5)	24.1(20.3)	56.1(52.0)	38.9(33.2)	91.1(86.5)	40.2(35.9)
F/I	–47.8(−46.0)	18.7(15.2)	58.3(54.2)	35.6(30.2)	73.3(69.1)	26.3(22.5)
Cl/Br	–8.2(−7.7)	63.9(58.5)	92.0(86.8)	80.0(71.8)	104.3(99.4)	47.6(43.2)
Cl/I	–15.1(−14.3)	58.6(53.4)	92.9(87.7)	77.2(69.5)	93.3(88.7)	37.7(33.6)
Br/I	–6.9(−6.5)	69.3(63.6)	101.3(95.7)	87.2(78.6)	95.0(90.3)	37.5(33.4)

a In the case of identity reactions, X and Y are the same; otherwise, Y denotes the halogen with the higher atomic number.

**Table 2:** Classical and Zero-Point Vibrational Energy Corrected (Adiabatic) Energies of X^–^ + SiH_3_Y → P + Q (X, Y = F, Cl, Br, I) Reaction Channels Obtained at the CCSD(T)-F12b/aug-cc-pVQZ(-PP)//CCSD(T)-F12b/aug-cc-pVTZ(-PP) Level of Theory in kcal/mol^a^

Q	Y^–^	HX	H^–^	XHY^–^	XY	XY^–^
P	SiH_3_X	SiH_2_Y^–^	SiH_2_XY	^1^SiH_2_	SiH_3_^–^	SiH_3_
F/F	0.0(0.0)	4.2(2.6)	–3.2(−6.0)	43.4(39.8)	164.5(160.7)	126.5(123.2)
Cl/Cl	0.0(0.0)	31.9(29.0)	50.1(46.5)	56.0(50.1)	106.6(103.0)	81.7(78.9)
Br/Br	0.0(0.0)	37.8(34.6)	62.0(58.0)	58.2(52.4)	95.6(92.0)	67.4(64.8)
I/I	0.0(0.0)	41.5(38.0)	74.3(70.1)	60.6(54.5)	81.0(77.5)	53.2(50.8)
F/Cl	–48.3(−47.6)	–8.0(−9.2)	–0.1(−2.9)	16.0(14.1)	97.7(94.4)	76.7(74.0)
F/Br	–59.4(−58.3)	–12.7(−13.7)	0.9(−1.9)	8.4(6.7)	82.1(79.0)	61.2(58.8)
F/I	–71.1(−69.7)	–18.5(−19.4)	2.3(−0.6)	0.3(−1.1)	59.6(56.7)	42.6(40.4)
Cl/Br	–11.1(−10.8)	27.2(24.5)	50.6(47.0)	49.6(45.3)	95.3(91.9)	68.6(66.0)
Cl/I	–22.7(−22.2)	21.3(18.8)	51.3(47.7)	42.0(38.2)	79.5(76.3)	53.9(51.6)
Br/I	–11.7(−11.4)	32.0(29.0)	62.5(58.5)	51.9(47.2)	81.3(77.9)	53.8(51.4)

a In the case of identity reactions, X and Y are the same; otherwise, Y denotes the halogen with the higher atomic number.

For the X^–^ + SiH_3_Y reactions, the S_N_2 channels are even more exothermic than the C-centered analogues. Here, proton abstraction is more favored than halogen abstraction in all cases. Moreover, proton abstraction is always the lowest-energy above S_N_2, except for X/Y = F/F, where hydride substitution is exothermic becoming even more favored than the thermoneutral S_N_2 channel. Furthermore, proton abstraction is also exothermic for X = F and Y = Cl, Br, I, though in these cases, S_N_2 is even more exothermic. Unlike for the C-centered systems, XY^–^ + SiH_3_ formation has always significantly higher reaction enthalpy than proton abstraction and in all cases, except for I/I, both hydride substitution and XH···Y^–^ formation are more favored than halogen abstraction. The XH···Y^–^ + ^1^SiH_2_ channel is above the H^–^ + SiH_2_XY asymptote for X/Y = F/F, Cl/Cl, F/Cl, F/Br and in all other cases, the former is less endothermic than the latter. (Note that unlike in the case of CH_2_, singlet is the ground electronic state of SiH_2_.) XY + SiH_3_^–^ is always the most endothermic channel, which corresponds to an excited electronic state similar to the carbon-centered analogue.

### 3.6 Basis Convergence

**Figure 9:**
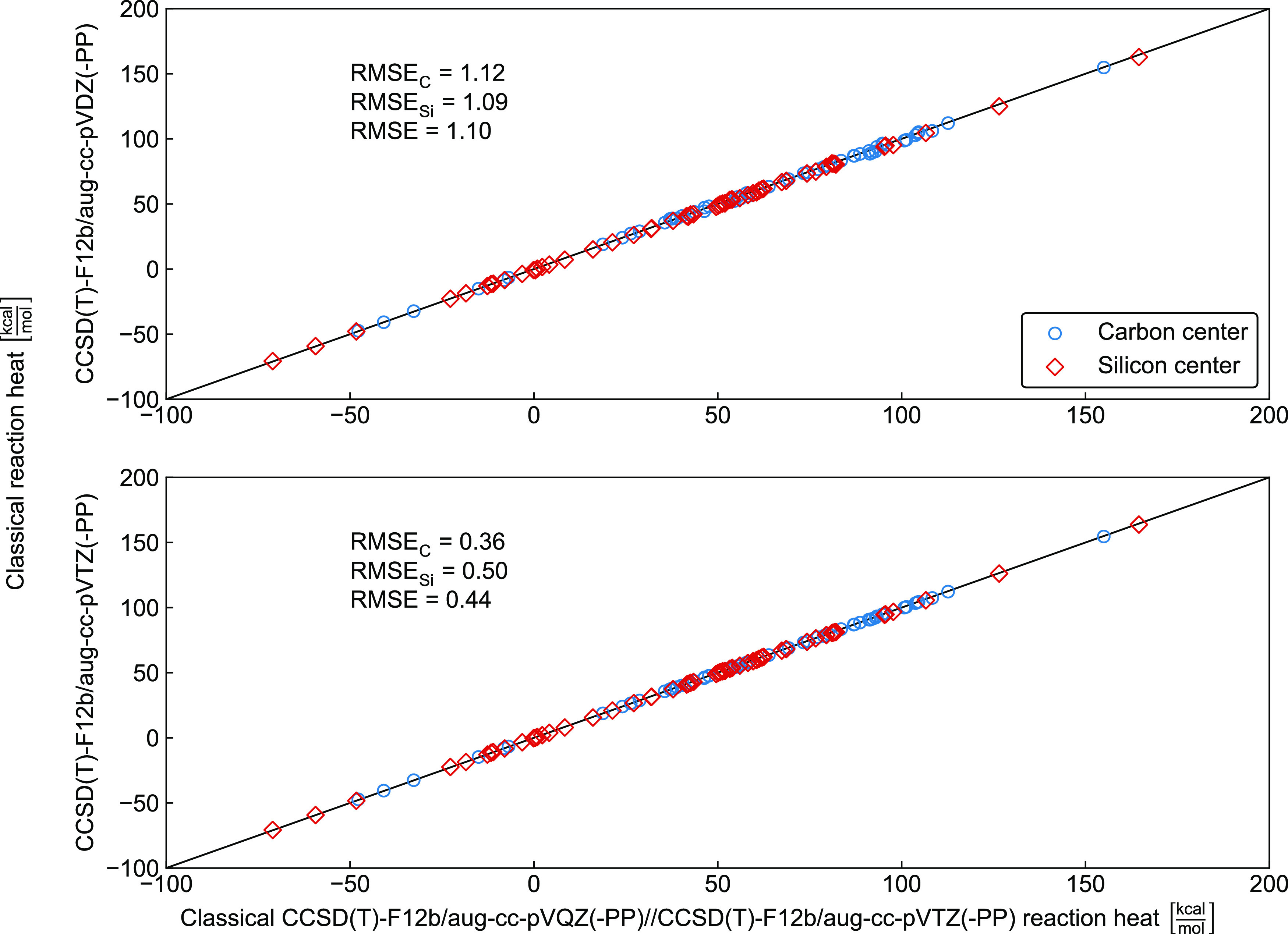
Comparison of classical reaction heats of X^–^ + AH_3_Y → P + Q (X, Y = F, Cl, Br, I; A = C, Si; Q/P = Y^–^/AH_3_X, HX/AH_2_Y^–^, H^–^/AH_2_XY, XHY^–^/AH_2_, XY/AH_3_^–^, XY^–^/AH_3_) reactions obtained at the CCSD(T)-F12b/aug-cc-pV*n*Z(-PP) (*n* = D, T) and CCSD(T)-F12b/aug-cc-pVQZ(-PP)//CCSD(T)-F12b/aug-cc-pVTZ(-PP) levels of theory showing carbon-centered (RMSE_C_), silicon-centered (RMSE_Si_), and combined (RMSE) root-mean-square errors in kcal/mol.

**Figure 10:**
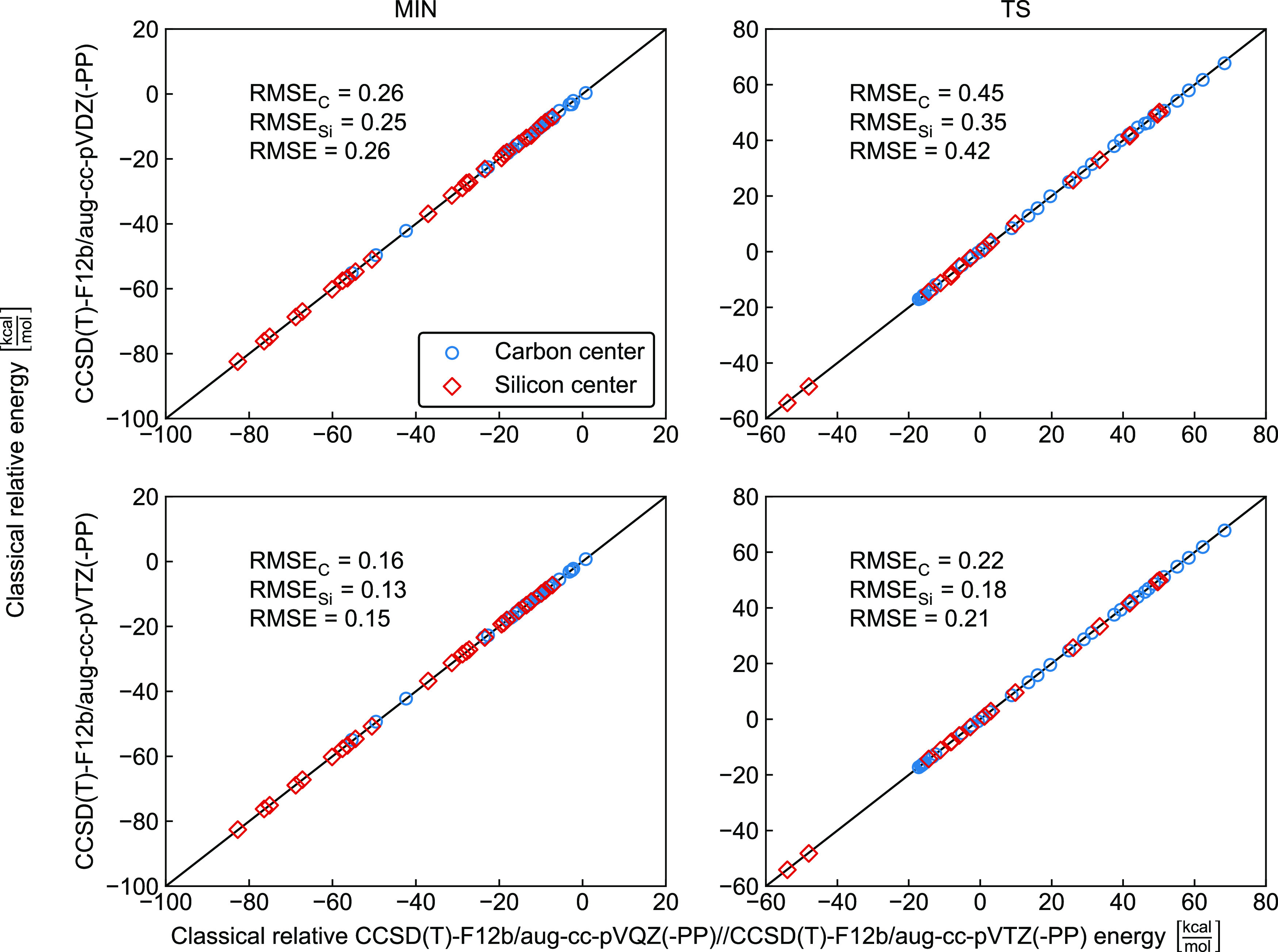
Comparison of classical relative energies of minima and saddle points in the interaction regions of the X^–^ + AH_3_Y (X, Y = F, Cl, Br, I; A = C, Si) S_N_2 reactions obtained at the CCSD(T)-F12b/aug-cc-pV*n*Z(-PP) (*n* = D, T) and CCSD(T)-F12b/aug-cc-pVQZ(-PP)//CCSD(T)-F12b/aug-cc-pVTZ(-PP) levels of theory showing carbon-centered (RMSE_C_), silicon-centered (RMSE_Si_), and combined (RMSE) root-mean-square errors in kcal/mol.

We studied the basis-set convergence of the CCSD(T)-F12b relative energies of all of the product channels and stationary points considered in the present study. The correlations of the aug-cc-pVDZ and aug-cc-pVTZ relative energies with the aug-cc-pVQZ reference data are shown in Figures 9 and 10 for the products and stationary points in the interaction region, respectively. For classical reaction heats, the aug-cc-pVDZ and aug-cc-pVTZ results agree with the aug-cc-pVQZ data with root-mean-square errors (RMSEs) of 1.10 and 0.44 kcal/mol, respectively. For the minima in the interaction regions, the corresponding RMSEs are 0.26 and 0.15 kcal/mol, whereas for the transition states the RMSE values are 0.42 and 0.21 kcal/mol, in order. Thus, one can see the fast basis-set convergence of the CCSD(T)-F12b method, often resulting in chemical accuracy even with the small aug-cc-pVDZ basis set. The larger RMSEs for the reaction enthalpies than for the S_N_2 stationary points in the interaction region can be explained by the fact that the reaction enthalpies cover a significantly wider energy range than the relative energies of the S_N_2 stationary points and the electronic structures of the reactants and S_N_2 stationary points are more similar than those of the reactants and products. Furthermore, larger transition-state RMSEs relative to those of the minima can also be understood knowing that the basis sets and ab initio methods usually perform better for minimum structures than for the electronically more problematic transition-state regions. Finally, comparing the basis-set convergence for the C- and Si-centered systems, only subtle difference can be seen as also shown in Figures 9 and 10.

## 4 Summary and Conclusions

Based on the recent knowledge accumulated on the reaction pathways of carbon-centered S_N_2 reactions,^16,19,25,30,32^ we present a comprehensive stationary-point characterization for the X^–^ + CH_3_Y and X^–^ + SiH_3_Y [X, Y = F, Cl, Br, I] S_N_2 reactions using the explicitly correlated CCSD(T)-F12b method with the correlation-consistent aug-cc-pV*n*Z [*n* = D, T, Q] basis sets. Basis-set convergence tests are carried out, and the results show that the relative energies obtained in the present study are usually well within chemical accuracy. The potential energy surfaces of the C- and Si-centered S_N_2 reactions are qualitatively different from several aspects. For C-centered reactions, the Walden-inversion process occurs on a double-well potential, where a central or a somewhat reactant-like transition state separates pre- and postreaction *C*_3*v*_ ion-dipole complexes. Furthermore, halogen-bonded *C*_3*v*_ front-side complexes and X = F hydrogen-bonded *C_s_* complexes and transition states also exist in the entrance channels. Moreover, for X/Y = F/I, the front-side complex and, for F/Cl and F/Br, the hydrogen-bonded complexes correspond to the deepest minimum in the entrance channel. In the case of the Si-centered reactions, the Walden inversion proceeds on a single-well potential via a Walden TS-like central or somewhat product-like minimum and hydrogen- and halogen-bonded complexes cannot be found. The front-side attack retention pathways have high barriers at C centers, whereas the front-side attack transition states are submerged in most cases at Si centers. Unlike for the C-centered reactions, at the Si center, front-side minima with X–Si–Y angles close to 90° are found both in the entrance and exit channels. For the first time, we report double-inversion transition states for Si-centered S_N_2 reactions, which are always above the reactant asymptotes and the front-side attack transition states. Thus, for Si-centered S_N_2 reactions, front-side attack is clearly the dominant retention mechanism, which could be competitive with Walden inversion in some cases. At C centers, clearly Walden inversion is the major S_N_2 mechanism in all cases and double inversion is the lowest-energy retention pathway for X = F.

In addition to the S_N_2 pathways, we also determine the reaction enthalpies of various additional product channels, including proton abstraction, hydride substitution, XH···Y^–^ formation, and halogen abstraction. These additional channels are always, usually highly, endothermic for the C-centered reactions, whereas at the Si centers, proton abstraction, hydride substitution, and XH···Y^–^ formation are exothermic for some X = F cases. The knowledge of the reaction enthalpies of the various possible product channels could be useful for future global potential energy surface developments for these systems. Furthermore, as all of the different product channels produce different ions, the experimental detection of these reactions could be straightforward. The present results could guide and motivate such experiments and reaction dynamics simulations to uncover the central atom effects in ion–molecule reactions.

## Supporting Information Available

Stationary point energies (XLSX)
Stationary point wavenumbers (XLSX)
Product energies (XLSX)
Product wavenumbers (XLSX)
Carbon-centered identity stationary points (TXT)
Carbon-centered nonidentity stationary points (TXT)
Silicon-centered identity stationary points (TXT)
Silicon-centered nonidentity stationary points (TXT)
Hydride substitution P C (TXT)
Hydride substitution P Si (TXT)
Proton abstraction P C (TXT)
Proton abstraction P Si (TXT)
Proton abstraction Q (TXT)
S_N_2 P C (TXT)
S_N_2 P Si (TXT)
XHY^–^ formation P C (TXT)
XHY^–^ formation P Si (TXT)
XHY^–^ formation Q (TXT)
XY^–^ anion formation P C (TXT)
XY^–^ anion formation P Si (TXT)
XY^–^ anion formation Q (TXT)
XY formation P C (TXT)
XY formation P Si (TXT)
XY formation Q (TXT)

## Notes

Supporting Information: Cartesian coordinates, absolute energies, and vibrational frequencies of the stationary points for the X^–^ + AH_3_Y → P + Q (X, Y = F, Cl, Br, I; A = C, Si) reactions, where P denotes the product with the central atom, C or Si.
